# Identification of a Bacterial-Like HslVU Protease in the Mitochondria of *Trypanosoma brucei* and Its Role in Mitochondrial DNA Replication

**DOI:** 10.1371/journal.ppat.1000048

**Published:** 2008-04-18

**Authors:** Ziyin Li, Megan E. Lindsay, Shawn A. Motyka, Paul T. Englund, Ching C. Wang

**Affiliations:** 1 Department of Pharmaceutical Chemistry, University of California, San Francisco, California, United States of America; 2 Department of Biological Chemistry, Johns Hopkins University School of Medicine, Baltimore, Maryland, United States of America; Washington University School of Medicine, United States of America

## Abstract

ATP-dependent protease complexes are present in all living organisms, including the 26S proteasome in eukaryotes, *Archaea*, and *Actinomycetales*, and the HslVU protease in eubacteria. The structure of HslVU protease resembles that of the 26S proteasome, and the simultaneous presence of both proteases in one organism was deemed unlikely. However, *HslVU* homologs have been identified recently in some primordial eukaryotes, though their potential function remains elusive. We characterized the HslVU homolog from *Trypanosoma brucei*, a eukaryotic protozoan parasite and the causative agent of human sleeping sickness. TbHslVU has ATP-dependent peptidase activity and, like its bacterial counterpart, has essential lysine and N-terminal threonines in the catalytic subunit. By epitope tagging, TbHslVU localizes to mitochondria and is associated with the mitochondrial genome, kinetoplast DNA (kDNA). RNAi of TbHslVU dramatically affects the kDNA by causing over-replication of the minicircle DNA. This leads to defects in kDNA segregation and, subsequently, to continuous network growth to an enormous size. Multiple discrete foci of nicked/gapped minicircles are formed on the periphery of kDNA disc, suggesting a failure in repairing the gaps in the minicircles for kDNA segregation. TbHslVU is a eubacterial protease identified in the mitochondria of a eukaryote. It has a novel function in regulating mitochondrial DNA replication that has never been observed in other organisms.

## Author Summary

ATP-dependent protein-hydrolyzing enzyme complexes are present in all living organisms, including the 26S proteasome in eukaryotes and the HslVU complex in bacteria. A simultaneous presence of both complexes in an organism was originally deemed unlikely until some *HslVU* homologs were found in certain ancient eukaryotes, though their potential function in these organisms remains unclear. We characterized an HslVU complex in *Trypanosoma brucei*, a protozoan parasite that causes human sleeping sickness in Africa. The complex is an active enzyme localized to the mitochondria of the parasite and closely associated with the mitochondrial DNA complex, which consists of several thousand small circular DNAs and a few dozen mitochondrial genomic DNAs. Depletion of this HslVU from the parasite resulted in a continuous synthesis of the small circular DNA, which led to aberrant segregation and incessant growth of the mitochondrial DNA complex to an enormous size that eventually blocks cell division. This novel HslVU function, which has not been observed in other organisms previously, could be a potential target for anti-sleeping sickness chemotherapy.

## Introduction

ATP-dependent protease complexes include the proteasomes in eukaryotes, *Archaea* and *Actinomycetales* and the HslVU complex in eubacteria [1]–[3]. The proteasomes rid the cells of mis-folded proteins and control the levels of many regulatory proteins that fluctuate during the cell cycle.

The mammalian 26S proteasome is composed of a 20S catalytic particle (CP) capped at one or both ends with a 19S regulatory particle (RP). The 20S CP is composed of 7 distinct α-subunits and 7 distinct β-subunits. Three catalytic β-subunits each having an N-terminal threonine and a lysine at position 33 are playing essential roles for activity [1]. The 19S RP binds, unfolds, and translocates polyubiquitinated protein substrates into the interior of 20S CP, where proteolysis occurs [1]–[3].

In the HslVU protease from *Escherichia coli*, the HslV subunit has characteristics resembling those of the catalytic β-subunits of 20S CP with a similar fold and two N-terminal threonines plus a lysine #33 playing essential roles in catalysis [2], [4]–[6]. Both threonines are required for maximum enzyme catalysis, because mutation of the first threonine to serine or valine eliminated activity and a mutation of the second threonine reduced activity by 60–70% [6]. Two stacked hexameric rings of HslV, which are capped at one or both ends with a hexameric ring of the AAA-type ATPase HslU [4], form the proteolytic complex. Like the 19S RP, the HslU ring recognizes and unfolds protein substrates and translocates them into the HslV proteolytic chamber [3].

Bacterial HslVU is limited in function [7]. Its deletion inhibits growth and viability of *E. coli* only at higher temperatures [8]. HslV responds to heat shock by degrading the heat shock factor σ32 [9],[10] and the cell-division inhibitor SulA [8],[11].

The co-existence of a 26S proteasome with an HslVU protease in the same living organism was originally considered unlikely [2]. However, recent genomic data suggest that *Trypanosoma, Leishmania*, and *Plasmodium* [12],[13] as well as amoebozoa, plantae, chromoalveolata, rhizaria and excavata species [14] could contain both the 26S proteasome and HslVU protease. The latter could be associated with mitochondria due to the presence of putative mitochondrial targeting signals. Our interest in cell cycle regulation by proteasomes prompted us to examine the HslVU homolog in *Trypanosoma brucei*, a parasitic protozoan causing sleeping sickness in Africa. We found that knockdown of this protease by RNA interference (RNAi) has remarkable effects on the mitochondrial genome, known as kinetoplast DNA (kDNA). kDNA is a complex network consisting of several thousand minicircles and a few dozen maxicircles topologically interlocked and condensed into a disk-shaped structure closely associated with the extra-mitochondrial flagellar basal body [15],[16]. Maxicircles, encoding ribosomal RNA and some of the subunits of respiratory complexes, produce transcripts that are edited by inserting or deleting uridylate residues to form an open reading frame. Editing specificity is provided by guide RNAs, most of which are encoded by minicircles (For reviews, see [17]–[19]).

Replication of kDNA [17],[18] occurs nearly concurrently with nuclear S phase [20]. It involves a topoisomerase II-mediated release of covalently-closed minicircles from the network into the kinetoflagellar zone, a region between the kDNA disk and mitochondrial membrane near the flagellar basal body [21]. Proteins within the kinetoflagellar zone, including UMSBP (the minicircle origin recognition protein) [22] and two DNA polymerases [23], then trigger replication and probably segregation of the free minicircles. The progeny free minicircles are thought to migrate to the two antipodal sites [24], which are protein assemblies situated ∼180° apart on the periphery of the kinetoplast disk. Late stages of minicircle replication occur within the antipodal sites, including removal of minicircle replication primers by a structure-specific endonuclease I (SSE1) [25] and repair of most of the gaps by DNA polymerase beta [26] and DNA ligase [27]. Finally, a topoisomerase II [28], also in the antipodal sites, reattaches the still-gapped minicircles to the network periphery [29], thereby enlarging the network. This process continues until all minicircles have replicated. Then the gaps are repaired by a polymerase and a ligase within the kDNA disk [26],[27]. The double-sized network splits in two [30], and these progeny networks are pulled into the two daughter cells by their linkage to the segregating flagellar basal bodies through a transmembrane tripartite attachment complex [15],[16].

In this report we show that *T. brucei* HslVU is mitochondrial and enriched in the kinetoplast region. Knockdown of the protease causes over-replication of minicircles, resulting initially in abnormal kinetoplast segregation and ultimately in formation of giant kDNA networks. Our results show that this HslVU complex regulates replication of a mitochondrial genome.

## Results

### Identification of the HslVU genes in *T. brucei*

**Figure 1:**
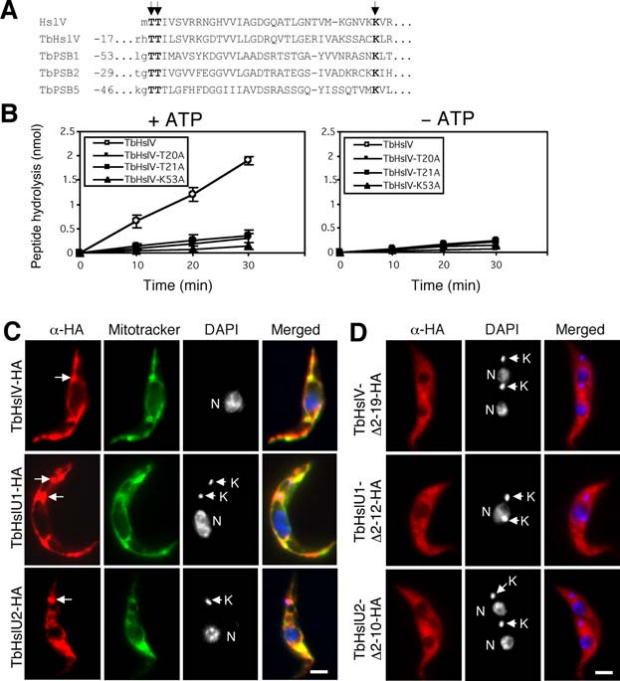
Enzymatic activity and intracellular localization of TbHslVU. (A). TbHslV contains the conserved threonine and lysine residues (arrows) found essential for the activities of HslV in *E. coli* [6] and the β-subunits of 20S CP in *T. brucei* [54]. (B). The ATP-dependent peptidase activity of TbHslV. Wild type and three TbHslV mutants T20A, T21A and K53A were expressed as HA-tagged proteins in *T. brucei*, immunoprecipitated and assayed for hydrolysis of Cbz-Gly-Gly-Leu-AMC. (C). Cells stably expressing TbHslVU-HA were labeled for mitochondria with Mitotracker green dye (green), immunostained with anti-HA mAb for HA-tagged proteins (red) and counterstained with DAPI for DNA (blue). Arrows indicate the focal points of HA-staining corresponding to the positions of kinetoplasts (arrowheads). (D). Cells expressing TbHslVU-HA with the putative mitochondrial targeting sequences deleted were stained with anti-HA antibody (red) and counterstained with DAPI. Bars: 2 µm.

We identified in the trypanosome genome database (www.genedb.org) an HslV homolog (Tb11.01.2000; designated TbHslV) with ∼40% identity to bacterial HslV (Fig. S1A) and a 15–24% overall identity to the three catalytic β-subunits in *T. brucei* 20S CP (data not shown). In addition, we found two HslU homologs, TbHslU1 (Tb927.5.1520) and TbHslU2 (Tb11.01.4050), that are 40–44% identical to *E. coli* HslU and ∼40% identical to each other (Fig. S2A). These proteins have potential N-terminal mitochondrial targeting signals. In addition, TbHslV has two threonines (T20 and T21) next to the targeting signal and a downstream lysine at position 53 (Fig. 1A, arrows). Both TbHslU1 and TbHslU2 possess the putative NTP-binding motif (P-loop) and the conserved residues essential for the ATPase activity of HslU (Fig. S2A, arrows). By homology modeling [31], TbHslV, TbHslU1 and TbHslU2 can be folded into three-dimensional structures resembling those of the HslV and HslU of *E. coli* (Figs. S1B and S2B; [4].

A Northern blot of total trypanosome RNA revealed that all the three genes are transcribed at comparable levels in both procyclic (insect) and bloodstream forms of *T. brucei* (data not shown). Furthermore, a Western blot showed that PTP-tagged TbHslV is expressed in procyclic trypanosomes (data not shown).

### The peptidase activity of TbHslV

We next tested whether TbHslV functions as a threonine peptidase and whether T20, T21, and K53 are essential for activity. We replaced each of these residues with alanine (Fig. 1A, arrows) in TbHslV tagged with a hemagglutinin (HA) epitope at the C-terminus. After expression in *T. brucei*, we immunoprecipitated each mutant protein (presumably in a complex with TbHslU1+2), and assayed for peptidase activity using Cbz-Gly-Gly-Leu-AMC as substrate. ATP-dependent peptidase activity was detected with wild type TbHslV, but it was strongly impaired by the mutations (Fig. 1B). It thus appears that these residues contribute to the peptidase activity of TbHslV.

### The subcellular localization of TbHslVU

The three TbHslVU subunits were predicted to be mitochondrial because of the targeting sequences predicted by the TargetP program (http://www.cbs.dtu.dk/services/TargetP/). To determine if they were indeed mitochondrial, we tagged each of them with a C-terminal HA epitope and expressed them in procyclic trypanosomes by tetracycline (0.1 µg/ml) induction. Immunofluorescence assay revealed a net-like distribution of the proteins that was closely associated with the mitochondrion stained by Mitotracker green (Fig. 1C). Deletion of the putative targeting sequence from each of the three proteins resulted in a failure to localize to the mitochondrion. Instead, they dispersed throughout the cytoplasm (Fig. 1D). TbHslVU immunofluorescence was often enriched in the kinetoplast region (see Fig. 1C and Figs. S3 and S4B), raising the possibility that its function may be related to kDNA.

### Effects of RNAi knockdown of TbHslVU

**Figure 2:**
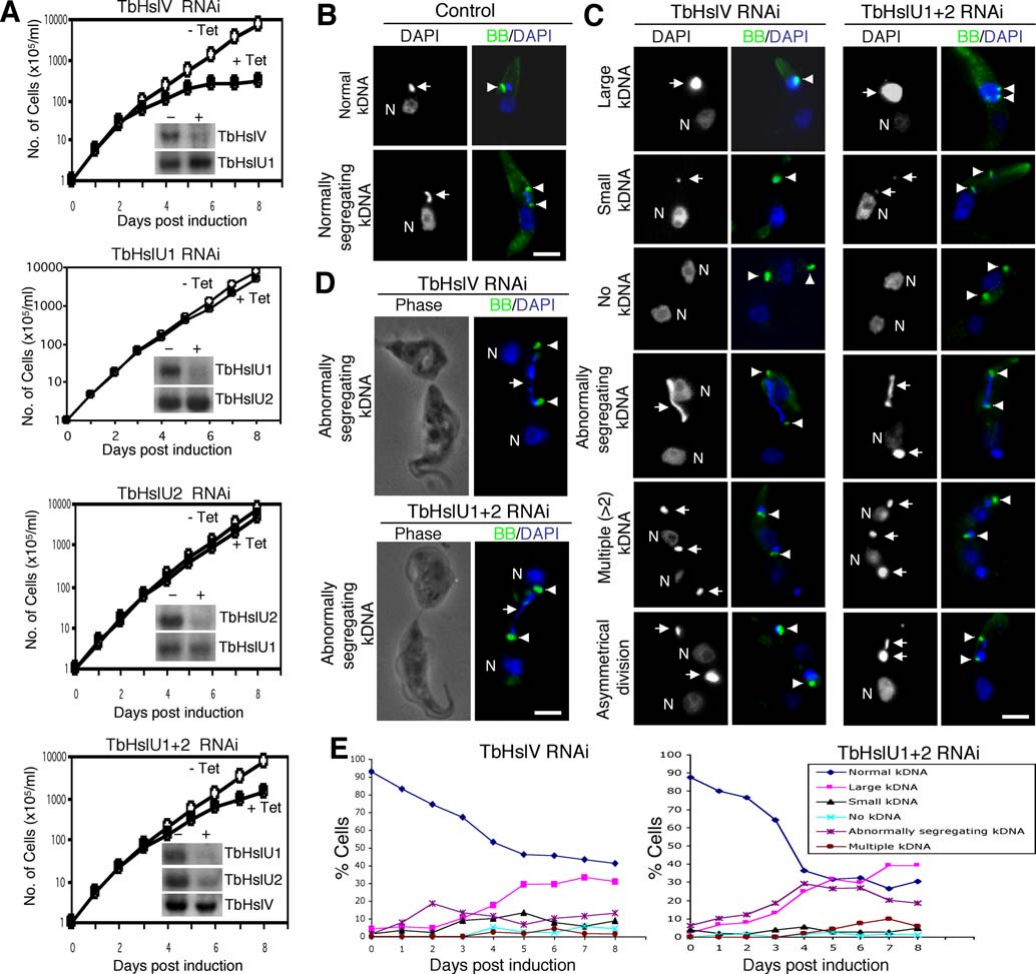
RNAi knockdown of TbHslVU affects cell growth and kinetoplast morphology. (A). Cells were grown in the presence (+) of tetracycline to induce RNAi for 7 days, and cell growth was monitored daily. Northern blots were performed to assess levels of TbHslV, TbHslU1 and TbHslU2 mRNA before (−) and after (+) 2 days of RNAi (insets). (B–E). Un-induced control cells (B) and cells after RNAi induction for 7 days (C) were labeled with YL1/2 antibody for basal bodies (BB, arrowheads) and counterstained with DAPI for nucleus (N) and kinetoplast (arrows). (D). Two TbHslVU RNAi cells at the final stage of cell division were still connected by a thin thread of kinetoplast DNA (arrows) between two basal bodies (arrowheads) in two well-separated cells. Bars: 2 µm. (E). Tabulation of RNAi cells with kinetoplasts in varying sizes and morphologies. Approximately 200 cells were counted at each time point and the data represent averages from three independent experiments.

To evaluate the function of TbHslVU, we used RNAi to knock down expression of each of the three subunits in procyclic trypanosomes. Knockdown of individual transcripts, confirmed by Northern blots (Fig. 2A, insets), resulted in modest to strong growth inhibition. Knockdown of TbHslV registered the highest inhibitory effect (Fig. 2A). Simultaneous knockdown of TbHslU1 and TbHslU2 led to a larger growth defect than that from individual knockdowns, though still not as severe as that from a TbHslV knockdown (Fig. 2A). DAPI staining showed significant changes in the size and shape of kinetoplasts in the RNAi cells (Fig. 2C), suggesting that TbHslVU could be involved in controlling replication and/or segregation of the kDNA network.

Analysis of DAPI-stained cells showed that knockdown of TbHslVU for 7 days resulted primarily in either large kinetoplasts (Fig. 2C) or kinetoplasts undergoing abnormal segregation (Figs. 2C and 2D). Of the segregation defects, about 5–10% had cells undergoing asymmetric division of the kinetoplast (Fig. 2C), and others, constituting about 15–20% of the total, had the two kinetoplasts joined by a thick thread of DAPI-stained material up to ∼5.6 µm in length (see Fig. 2C under abnormally segregating kDNA). In contrast, the normally segregating kinetoplast in the control cell has an estimated length of ∼1.4 µm (see Fig. 2B). A third form, representing ∼1% of the total, has the nucleus and basal body already divided and segregated into two sister cells, but there appeared to be incomplete segregation of the kinetoplast. The single kinetoplast was stretched out through its central region and situated within an intercellular bridge at the posterior ends of the two divided cells, which were separated by a distance of ∼6.1 µm (Fig. 2D). Only a few cells were found with small kinetoplasts, multiple (>2) kinetoplasts, or none at all (see examples in Figs. 2C and 2D). Fig. 2E shows kinetics of appearance of the aberrant forms of kinetoplast as a function of time after RNAi. By the end of the experiment, the majority of the cells had abnormal kinetoplasts, though 30 to 40% still appeared normal.

### RNAi of TbHslVU resulted in a selective increase of kDNA minicircles and an increase of kDNA network size

**Figure 3:**
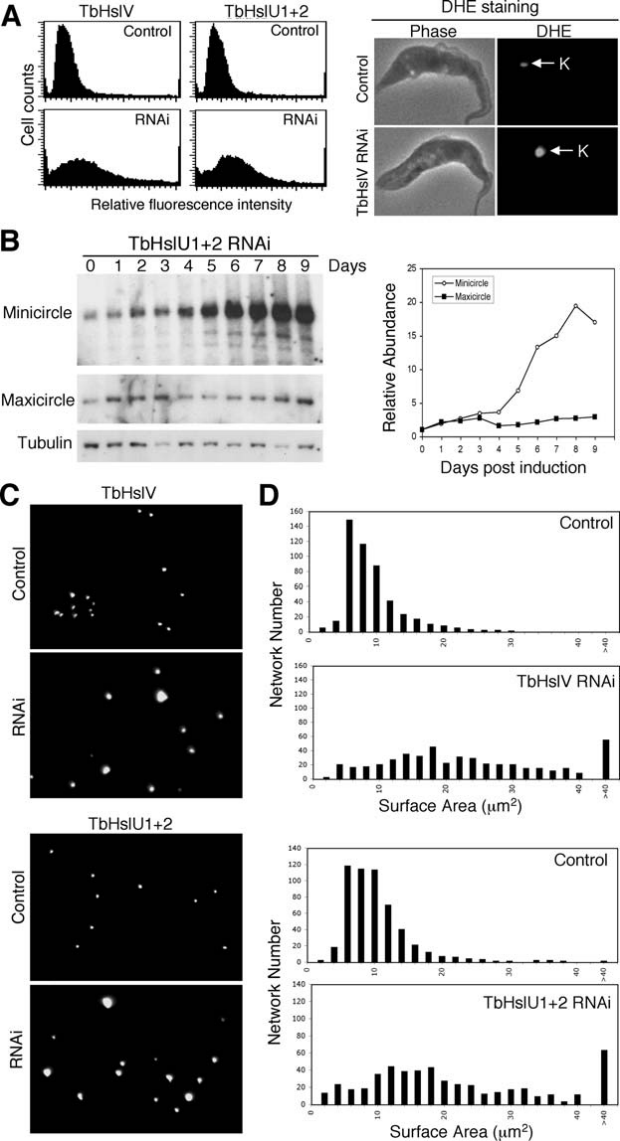
TbHslVU RNAi led to heterogeneously sized kinetoplasts. (A). Flow cytometry analysis of DHE stained cells. A total of 25,000 cells were counted in each experiment (Left panel). DHE stains exclusively the kinetoplasts in control and RNAi cells (Right panel). (B). Southern analysis of changes in minicircle and maxicircle DNA content during TbHslU RNAi. The kinetics of minicircle (open circle) and maxicircle (filled square) accumulation are presented to the right of the Southern blots. (C). DAPI staining of the isolated kDNA networks. (D). Surface areas of the isolated kDNA networks stained with DAPI, and measured with the NIH Image software.

As another approach to assess kinetoplast size, we used dihydroethidium (DHE) that selectively stains the kDNA but not the nuclear DNA (Fig. 3A, Right panel) (DHE is oxidized to ethidium in the mitochondrion but not in the nucleus, thus staining only kDNA [32]). By flow cytometry, the DHE-stained TbHslV and TbHslU1+2 knockdown cells (7 days after RNAi) had a much broader distribution of fluorescence with higher intensity than that of the control cells (Fig. 3A, Left panel), indicating that the average kDNA/cell increases following RNAi.

To determine whether the kDNA increase involved minicircles, maxicircles, or both, we isolated total DNA after RNAi, digested the time samples with restriction enzymes and, after gel electrophoresis, probed a Southern blot for minicircles and maxicircles. We found that minicircle DNA increased significantly (∼15–20 fold) in TbHslU1+2 RNAi cells after 7 days, whereas maxicircle DNA increased only ∼2.8 fold (Fig. 3B). Thus, RNAi of TbHslU has a much greater effect in enhancing the level of minicircles.

This increase in minicircles (Fig. 3B) could be attributed to either an enlarged kDNA network or the presence of multiple closely-packed networks. To distinguish between the two possibilities, kDNA networks were isolated from 7-day knockdown cells, stained with DAPI, and their surface areas were measured. Networks from control cells were small in size (Fig. 3C) with a peak representing an average surface area of ∼8 µm^2^ (Fig. 3D). In contrast, networks from RNAi cells had a much broader size distribution ranging from 2 to over 40 µm^2^ (Fig. 3D) with the largest network exceeding ∼215 µm^2^. These data prove that the increased kinetoplast size and minicircle level are primarily due to enlargement of the network and not to an increased number of normal-sized networks.

**Figure 4:**
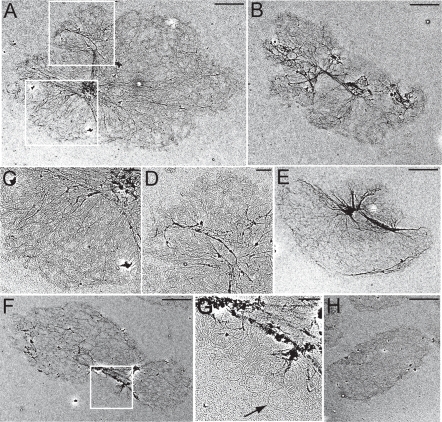
Electron microscopic examination of kDNA networks from the control and TbHslV RNAi cells. Methods were described by [38]. (A, B, E, F) kDNA networks from cells after 7 days of TbHslV RNAi. (H) A kDNA network from an un-induced control cell. (C, D) Enlargements of the network in A, corresponding to the areas outlined in white framed boxes. (G) Enlargement of the network in F framed in a white box. The arrow in G indicates a maxicircle. Scale bars for A, B, E, F, and H, 2 µm and for C, D, and G, 0.5 µm.

This conclusion was confirmed by EM of isolated networks. Fig. 4H shows a network from a control cell with a typical elliptical shape and planar structure; it is about ∼6 µm in length and ∼3 µm in width, a standard size of kinetoplast after being processed for electron microscopy. Those from cells after 7 days of TbHslV RNAi were grossly enlarged, heterogeneous in size and irregular in shape with estimated lengths ranging from ∼10 µm to ∼16 µm (Figs. 4A, 4B, 4E and 4F). Electron-dense fibers were present in these enlarged networks. The one in Fig. 4F, apparently undergoing asymmetrical division, has, like the wild type, a cluster of maxicircles located between the two lobes (Fig. 4G).

### Effect of RNAi on free minicircle replication intermediates

**Figure 5:**
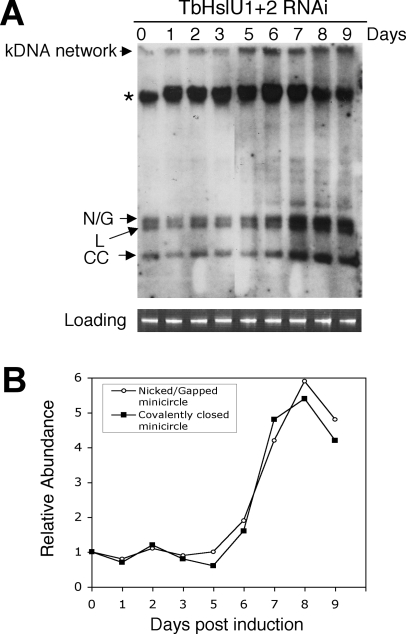
Effect of TbHslU1+2 double RNAi on free minicircle replication intermediates. (A). Total DNA was fractionated on an agarose/ethidium gel and a Southern blot was probed for minicircles. N/G, nicked/gapped minicircles; L, linearized minicircles; CC, covalently-closed minicircles, *, nonspecific hybridization to nuclear DNA. The nicked/gapped minicircles form a doublet with the lower component possibly linearized minicircles. Since it is present prior to RNAi induction (day 0), it is likely unrelated to RNAi. (B). Quantitation (by Phosphorimager) of bands from A. The nicked/gapped species includes both components of the doublet.

To analyze the effect of TbHslVU RNAi on the free minicircle species, we fractionated total DNA from control and TbHslU1+2 RNAi cells on an agarose gel in the presence of ethidium bromide to resolve covalently-closed free minicircles from those containing gaps [29]. Probing a Southern blot for minicircles revealed that the levels of covalently-closed and gapped free minicircles remained constant during the first 5 days of RNAi and then dramatically increased by 5 to 6-fold by the end of the 9 day experiment (Fig. 5), implying that silencing of TbHslU enhances the rate of minicircle replication.

### RNAi of TbHslVU alters the distribution of gapped circles in kDNA

**Figure 6:**
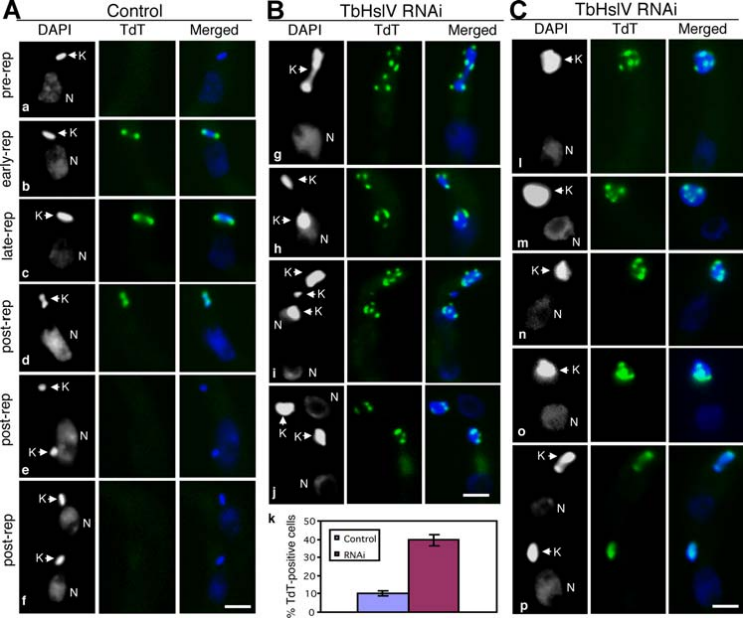
*In situ* TdT-catalyzed Fluorescein-dUTP labeling in cells after 7 days of TbHslV RNAi. (A). The control cells (a–f). (B, C). The TbHslV RNAi cells (g–p). Percentages of TdT-labeled cells in control and TbHslV RNAi cells are presented (k). Bars: 2 µm.

To investigate the organization of the replicating kinetoplast in RNAi cells, we *in situ* labeled gapped minicircles (and maxicircles) at 3′-OH groups using terminal deoxynucleotidyl transferase (TdT) and fluorescent deoxyuridine triphosphate [33],[34]. In control cells, we detected no TdT labeling of kDNA before kinetoplast replication as all minicircles are covalently closed (Fig. 6A, a). At the early stage of kinetoplast replication, there is strong TdT labeling at the two antipodal sites enriched in multiply-gapped free minicircles, not yet attached to the network (Fig. 6A, b). At the late stage of replication, when many gapped minicircles had attached to the network, TdT-label is still strong in the antipodal sites, but the network, especially the polar regions, are also labeled weakly because they contain minicircles which had most but not all of their gaps repaired just prior to network attachment (Fig. 6A, c). When the kinetoplast was undergoing segregation, TdT label spread over the entire network (Fig. 6A, d) until the completion of segregation when all the minicircles became covalently closed and could no longer be labeled (Fig. 6A, e–f).

We observed a completely different pattern of TdT-labeling in TbHslV RNAi cells. As shown in Fig. 6B, k, the frequency of TdT-labeling increased to ∼40% of the cells after 7 days. About 95% of the RNAi cells with enlarged or abnormally segregating kinetoplasts were TdT-positive (Fig. 6B, k). As for the pattern of TdT-lableing of TbHslV-deficient cells, there were 3 distinct categories. First, ∼26% of the kinetoplasts contained multiple bright TdT-labeled dots, most of which appeared on the periphery. They number up to 7 in abnormally segregating kinetoplasts (Fig. 6B, g) and from 3 to 6 in enlarged kinetoplasts (Fig. 6B, h–j). The second category, constituting ∼6% of the cells, had the TdT labeled dots but also had a diffuse background of TdT labeling (Fig. 6C, l–m). The third category, with ∼8% of the cells, had large regions or all of the kinetoplast uniformly stained (Fig. 6C, o–p), although TdT labeling appeared punctate in Fig. 6C, o, as if the TdT dots are merging together. In contrast to control cells, TdT labeling was still detectable in kinetoplasts after segregation (Fig. 6B, h–j, Fig. 6C, p with Fig. 6A, e–f).

### Localization of minicircles and maxicircles by FISH

**Figure 7:**
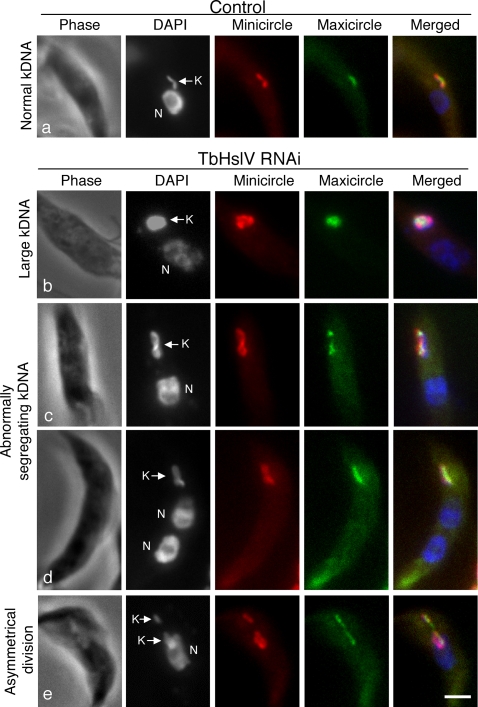
*In situ* detection of minicircles and maxicircles in the kinetoplast by FISH. Cells were fixed, probed for minicircles (red) or maxicircles (green), and counterstained with DAPI. kDNA is indicated with a K and nucleus with an N. Bar: 2 µm. FISH does not detect covalently-closed DNA minicircles because they are non-denaturable [24]. This is likely also true for maxicircles. Thus the FISH signal may not be proportional to the total populations of minicircles and maxicircles. Bars: 2 µm.

We used fluorescence *in situ* hybridization (FISH) to investigate the effect of TbHslV RNAi on distribution of minicircle and maxicircle DNAs within the network [24]. In control cells, the late-replicating network has the minicircles organized in a dumbbell shape with maxicircles clustered in the middle (Fig. 7a; see also [30], [35]–[37]). In an enlarged kinetoplast from a TbHslV RNAi cell, however, minicircle DNA was spread out over nearly the entire DAPI-stained network, whereas maxicircle DNA remained at the center (Fig. 7b). In abnormally segregating kinetoplasts, the minicircles also filled most of the DAPI-stained network, whereas the maxicircle was segregated into two symmetrical clusters (Fig. 7c). Due to the excessive size of minicircle network, it turned into a thread between the segregated maxicircles. Of 30 abnormally segregating kinetoplasts, 23 had segregated their maxicircles, in striking contrast to those in the control cells, which remained in the center (compare Figs. 7c and 7a). In the remaining 7, maxicircles have not segregated and appeared like the control (compare Figs. 7d and 7a). In the asymmetrically dividing kinetoplast, minicircle DNA was also distributed unevenly, constituting the basis of unevenly sized kinetoplasts, whereas maxicircle DNA was always symmetrically segregated (Fig. 7e). These results suggest that uneven segregation and enlargement of the kinetoplasts can be attributed to the excessive synthesis and uneven distribution of minicircles.

### Expression of peptidase-dead TbHslV mutants led to a partial dominant-negative effect

Expression of the peptidase-dead TbHslV mutants (TbHslV-T20A, TbHslV-T21A and TbHslV-K53A) in *T. brucei* (see Fig. 1B) also led to reduced cell growth (Fig. S4A), but the extent of reduction was less significant than that by a TbHslV knockdown, presumably due to the fact that wild type TbHslV was also expressed in these cells (compare Fig. S4A with Fig. 2A). The mutant proteins localized to the mitochondria with an apparent enrichment in the kinetoplast like the wild type protein (Fig. S4B). Cells expressing the mutant proteins showed also enlarged or abnormally segregating kinetoplasts (Fig. S4B), similar to those from knocking down TbHslV (Fig. 2C). Thus, expression of the inactive TbHslV mutants generated a partial dominant-negative effect on kinetoplast replication and segregation. A likely formation of TbHslVU complexes of mixed compositions of wild type and mutant proteins could result in the partially reduced activity of TbHslVU.

## Discussion

We report here the identification of an HslVU protease, previously found only in prokaryotes, in a eukaryote. We demonstrated that *T. brucei* expresses an enzymatically active ATP-dependent HslVU homolog that localizes to the mitochondrion. More importantly, we discovered that the function of TbHslVU is to control replication/segregation of kDNA, the trypanosome mitochondrial genome.

RNAi of TbHslVU in its early stages had two major effects. First, it caused an increase in cells with kinetoplasts undergoing abnormal segregation. Second, it caused the appearance of giant kinetoplasts (Fig. 2C). Kinetic studies (Fig. 2E) showed that RNAi caused an initial increase in the abnormally segregating forms, followed by a decline. Then there was an increase in cells with enlarged kinetoplasts. The switchover from abnormally segregating forms to enlarged kinetoplasts occurred earlier by about 2 days when RNAi targeted TbHslV than when it targeted TbHslU1+2, presumably because RNAi is more effective in the former case (see Fig. 2A). The phenotype of HslVU knockdowns, due to over-replication of minicircles, has no mechanistic similarity to those of two other recently described RNAi cell lines that are also defective in segregation and that produce giant networks. In the case of p166 RNAi, these phenotypes develop because this protein is a component of the tripartite attachment complex [38]. Regarding UMSBP, RNAi not only affects kDNA segregation but also nuclear division and separation of the basal bodies [39].

It is possible that minicircle over-replication starts soon after induction of RNAi, resulting in a gradual increase in the size of the network. These large networks probably can segregate in the initial phase of RNAi, but because they are somewhat oversized, the segregation machinery may be unable to handle them properly. Thus they either undergo asymmetric division or produce some of the abnormal forms shown in Figs 2C and 2D. The increase in cells undergoing abnormal kinetoplast segregation (Fig. 2E) is probably because abnormal segregation may take longer than usual and thus a larger fraction of the cells are involved with this process at any given time. We speculate that as kDNA over-replication continues, the segregation machinery is blocked, and the network just continues to grow without segregation. It would be theoretically possible that the cells with large kinetoplasts could undergo cytokinesis, generating one cell with a large kDNA and another without any kDNA at all, as is the case following RNAi of p166 [38]. However, few cells without kDNA appear after RNAi of TbHslVU (Fig. 2E). Therefore, as is occurring following RNAi of TbHslV for 5 to 6 days (Fig. 2A), cell division slows down and eventually stops.

There is considerable evidence to support this model. Measurement of total minicircles reveals a ∼20-fold increase during TbHslVU RNAi (Fig. 3B), whereas maxicircles increase only 2.8-fold. The minicircle increase is biphasic, with the increased rate occurring at about the time when the number of abnormally segregating forms is declining (Fig. 2E). It is possible that once the network has become too large to segregate, it could spend a longer portion of the cell cycle undergoing replication, thus accounting for its accelerated growth in size. It is not surprising that the increased level of minicircles is accompanied by a corresponding increase in free minicircle replication intermediates (Fig. 5).

The mechanism by which minicircles over-replicate is not clear. It is thought that the gaps in minicircles are markers to distinguish those that have undergone replication from those that have not, thus ensuring that they replicate only once per generation [18]. This highly organized system appears to have broken down in cells in which TbHslVU has been knocked down. Since about 95% of the oversized kinetoplasts are TdT-positive, normal gap-repair mechanisms for network minicircles may have been overwhelmed by the accelerated rate of replication. Those that have their gaps repaired may be released from the network a second time, allowing the network to increase in size more than two-fold during one cell cycle.

The TdT labeling pattern of RNAi cells differ dramatically from those observed in kDNAs of other trypanosomatids. In *Crithidia fasciculata,* newly synthesized gapped minicircles are attached to the network adjacent to the antipodal sites, but then, due to rotation of the kinetoplast disk, they distribute around the network periphery; thus TdT labeling resembles a peripheral ring [37],[40]. In *T. brucei*, minicircles also attach to the network adjacent to the antipodal sites. However, instead of rotating, the disk oscillates, distributing minicircles in a limited region along the network periphery. Occasionally, there is a larger displacement of the kinetoplast, a jump, that moves the minicircle attachment site to a new position on the periphery where it resumes oscillation. Thus, due to a combination of oscillations and jumps, the gapped minicircle progeny accumulate at the two ends of the kinetoplast, accounting for the polar TdT labeling [37].

There is a completely different pattern of gapped minicircle distribution in TbHslV RNAi cells (Fig. 6). A predominant form has 3 to 6 dots of TdT label, mostly on the network periphery. This pattern implies that reattachment of gapped minicircles is not a random process and that there is still some order maintained in the replication of large networks. One possible explanation for the dot pattern is that the number of antipodal sites has increased so that each site is associated with a dot. During the kDNA replication cycle in normal cells, two new antipodal sites must assemble every generation [33]. In TbHslVU RNAi cells, the standard pair of antipodal sites may be unable to handle the 6-fold increase in free minicircle replication intermediates (Fig. 5), thereby additional pairs of antipodal sites are formed for the task. Another possibility is that the RNAi cells have only two antipodal sites, as in the wild type, but when functioning on a large network, jumps dominate over oscillations. After attachment of gapped minicircles at one site, forming a dot of TdT-label, a jump moves it to another site. Thus, two antipodal sites are able to create multiple TdT-labeled dots. There is precedent for RNAi changing the mechanism of minicircle attachment. RNAi of SSE1 changed the TdT labeling pattern from being polar to a ring. This pattern apparently was not due to rotation of the kDNA disk but to increasing the amplitude of oscillation to nearly 180° [37].

Although we do not have enough information to speculate further on the detailed mechanism of minicircle over-replication, we can conjecture about the role of TbHslVU in controlling the process. In principle, it could degrade some or all kDNA replication proteins when replication was complete, thereby stopping kDNA synthesis. Alternatively, TbHslVU could degrade only one protein, a master positive regulator of minicircle replication. The regulator cannot be degraded in TbHslVU RNAi cells, thus allowing kDNA replication to continue out of control. Possible candidates for this master regulator are the universal minicircle sequence binding protein (UMSBP) [41] and p38 [42], both of which bind the minicircle replication origin and could be involved in triggering their replication.

Since the function of TbHslVU in regulating DNA replication in *T. brucei* mitochondria has never been observed in bacteria, this distinction raises the interesting question of how this function was acquired. As discussed previously [43], it is thought that an ancestor of *T. brucei,* like present-day *Cryptobia helices,* had a mitochondrion that contained non-catenated plasmids, which encoded guide RNAs and had other minicircle-like properties. The pathway of kDNA evolution, leading to the network structure found in trypanosomatid parasites, was probably driven by a need to improve the accuracy of segregation of the multiple minicircle sequences required to encode the guide RNA repertoire. But the development of the network structure required a much more complex replication scheme. One example of complexity is that kDNA networks replicate during a discrete phase of the cell cycle, in contrast to mitochondrial DNAs in higher eukaryotes that replicate randomly throughout the cell cycle. One mechanism for this aspect of kDNA replication control could have involved recruitment of the HslVU homolog that had initially been acquired from the bacterial endosymbiont that formed the mitochondrion. In this regard, it will be very interesting to study the function of the HslVU homologs in other non-kinetoplastid eukaryotes.

## Materials and Methods

### *T. brucei* Cell Culture and RNA interference

The procyclic form of *T. brucei* strain 29-13 [44] was cultivated at 26°C in Cunningham's medium supplemented with 10% fetal bovine serum and 15 µg/ml G418 and 50 µg/ml hygromycin B.

The N-terminal coding regions of TbHslV, TbHslU1 and TbHslU2 were each cloned into pZJM vector [45] for RNAi. The TbHslU1 and TbHslU2 double knockdown construct was prepared by ligating the two fragments of TbHslU1 and TbHslU2 into the pZJM vector. The RNAi constructs were linearized and electroporated into *T. brucei* [46]. The transfectants were selected under 2.5 µg/ml phleomycin and cloned [47]. RNAi was induced by 1.0 µg/ml tetracycline to switch on the two opposing T7 promoters for dsRNA synthesis.

### Northern, Western and Southern Blots

Total RNA was blotted onto nitrocellulose membrane. Northern hybridization was carried out overnight at 42°C in 50% formamide, 6× SSC, 0.5% SDS, 5× Denhardt’s solution with 0.1 mg/ml salmon sperm DNA.

*T. brucei* cells were lysed and the lysate fractionated with SDS-PAGE, transferred onto PVDF membrane and immuno-blotted with anti-Protein C mAb that recognizes the PTP epitope tagged to TbHslV [48].

Total DNA was purified from trypanosome cells using the Cell Culture DNA Mini kit (Qiagen). For measurements of minicircle and maxicircle content, total DNA was digested with *Xba* I-*Hind* III, fractionated on a 1% agarose gel and transferred onto the nitrocellulose membrane. The membrane was then hybridized using fragments of minicircle conserved region, maxicircle or tubulin as probes [29].

To detect free minicircles, total DNA was fractionated on a 1% agarose gel with 1.0 µg/ml ethidium bromide, transferred onto a nitrocellulose membrane and hybridized with a minicircle probe.

### Kinetoplast DNA Staining, Flow Cytometry and Measurement of Network Surface area

Flow cytometry analysis of DHE-stained trypanosome cells was carried out as previously described [32] using a FACScan flow cytometer (Becton Dickinson Biosciences). Briefly, live cells were incubated with 10 µg/ml DHE (Molecular Probes) for 10 min at room temperature, washed once with PBS, and re-suspended in 1 ml PBS.

kDNA networks were isolated from control and RNAi cells [34], and stained with DAPI. The NIH Image software was used to measure the surface area of the planar structures in fluorescence micrographs (500 networks measured at each time point).

### Expression of Epitope-tagged Proteins

TbHslV, TbHslU1 and TbHslU2 were each tagged at the C-terminus with a triple HA epitope and cloned into a pLew100 vector [44]. The constructs were transfected into 29-13 cells. Stable transfectants were selected under 2.5 µg/ml phleomycin and cloned. TbHslV was cloned into the pC-PTP-NEO vector [48], which places the PTP-tagged TbHslV under the endogenous promoter, and transfected into the 427 cells. Stable transfectants were selected under 40 µg/ml G418.

### Peptidase Assay

Peptide hydrolysis was assayed as previously described [49]. Wild type and mutant TbHslV proteins were each immunoprecipitated with anti-HA mAb and protein A Sepharose CL-4B beads in the presence of 1 mM ATP, and incubated at 37°C for 30 min in the assay buffer containing 0.1 mM Cbz-Gly-Gly-Leu-AMC (Bachem). At different times, the reaction (100 µl) was terminated by adding 900 µl of 1% SDS, and the fluorescence of the reaction products was measured.

### Immunofluorescence Microscopy and Mitotracker Staining of Mitochondria

Cells were fixed with 4% paraformaldehyde and incubated with the primary antibodies at room temperature for 60 min, washed three times and incubated with FITC-conjugated or Cy3-conjugated secondary antibodies (Sigma-Aldrich) for another 60 min at room temperature. After three more washings, cells were mounted in Vectashield mount medium (Vector Labs, Inc.) containing 1.0 µg/ml of DAPI and examined with a fluorescence microscope. Anti-HA mAb was used for detecting the TbHslVU-HA fusion proteins. Rat monoclonal antibody YL1/2 and FITC-conjugated anti-rat IgG were used to label the basal bodies [50],[51].

*T. brucei* cells were incubated with 5 µM Mitotracker^TM^ green FM (Molecular Probes) for 20 min at 26°C, washed with fresh medium and incubated for another 20 min. The cells were then washed six more times with PBS, fixed and processed for immunofluorescence microspcopy.

### Fluorescent In Situ Hybridization (FISH)

The minicircle probe (73 nucleotides of the minicircle conserved region) was synthesized by PCR using isolated kDNA networks as template. The PCR DIG probe synthesis kit (Roche) was used to incorporate DIG-modified dUTP. The maxicircle probe was labeled by nick translation with biotin-modified dUTP (Roche) using standard protocols [52]. Templates for nick translation were plasmids pTKH128, pTKH38, and pTKHR34, a gift from Dr. Kenneth Stuart, together representing ∼80% of the maxicircle sequence [53]. The three maxicircle probes were pooled for a final concentration of 2.5 ng/µl in the hybridization experiments. FISH was performed as previously described [24].

### In Situ Labeling of kDNA Network with Fluorescein-dUTP Catalyzed by TdT

The nicks and gaps in minicircles were fluorescently labeled *in situ* with terminal deoxynucleotidyl transferase (TdT)-mediated dUTP nick end-labeling (TUNEL) using an *in situ* labeling kit (Roche) as previously described [33]. Cells were fixed in 4% paraformaldehyde and permeabilized in cold methanol. After re-hydration, cells were pre-incubated with labeling solution containing CoCl_2_, nucleotides, and Fluorescein-dUTP for 20 min at room temperature and then incubated for 60 min with the labeling solution containing TdT. The reaction was stopped with three washes in 2× SSC, 0.9× PBS and two washes in PBS. Samples were stained with DAPI and processed for fluorescence microscopy.

## Supporting Information

Figure S1 TbHslV resembles *E. coli* HslV protease. (A). Sequence alignment of TbHslV with HslV. Residues essential for the activity of HslV are indicated by arrows; (B). The homology model of TbHslV. Generation of the three-dimensional models was performed using Swiss-Model (http://swissmodel.expasy.org/) [31] according to the corresponding *E. coli* templates. The images were then analyzed with Swiss-Pdb-Viewer 3.7 (http://swissmodel.expasy.org/spdbv/). Protein Data Bank codes for the templates of HslV were 1ned [4], le94 [55] and 1hqy [56].
(1.21 MB TIF)

Figure S2 Both TbHslU1 and TbHslU2 resemble *E. coli* HslU. (A). Sequence alignment of TbHslU1, TbHslU2 with HslU. The NTP-binding domain (P-loop) is outlined and the residues important for HslU function are indicated by arrows; (B) The homology models of TbHslU1 and TbHslU2. Each structure was modeled on an *E. coli* HslU template. The three domains identified in HslU are also present in the two *T. brucei* homologs. The protein bank code for the template of HslU is 1do0 [4].
(3.28 MB TIF)

Figure S3 (A). Co-localization of TbHslVU proteins with the kinetoplast. Cells were labeled with anti-HA antibody for TbHslVU-HA (red), YL1/2 antibody for basal body (BB, green), and DAPI for nuclear (N) and kinetoplast (K) DNA. Arrows point to the bright spots of HA labeling, arrowheads point to the basal bodies, and open arrowheads indicate the co-localization of TbHslVU-HA protein with kinetoplasts. Bar: 2 µm. (B). Subcellular localization of TbHslV during different stages of kinetoplast cycle. Cells were labeled with anti-HA antibody for TbHslV-HA, and DAPI for nuclear (N) and kinetoplast (K) DNA. Arrows point to the bright spots of HA labeling, and open arrowheads indicate co-localization of TbHslV-HA with kinetoplasts. Bar: 2 µm.
(4.63 MB TIF)

Figure S4 Effects of expressing HA-tagged wild type and mutant TbHslV on cell growth (A) and kinetoplast segregation (B). Cells were labeled with anti-HA antibody (green) and counterstained with DAPI for the nucleus and kinetoplast. The arrows point to the bright spots of HA staining, the solid arrowheads point to the kinetoplasts and the open arrowheads indicate the HA spots superimposed with kinetoplasts. Bar: 2 µm.
(0.93 MB TIF)
