# Supplementary figures and images for: Identification of a Bacterial-Like HslVU Protease in the Mitochondria of Trypanosoma brucei and Its Role in Mitochondrial DNA Replication
Source: PLoS Pathog. 2008 Apr 18;4(4):e1000048. doi: 10.1371/journal.ppat.1000048 (PMC2277460; doi:10.1371/journal.ppat.1000048)

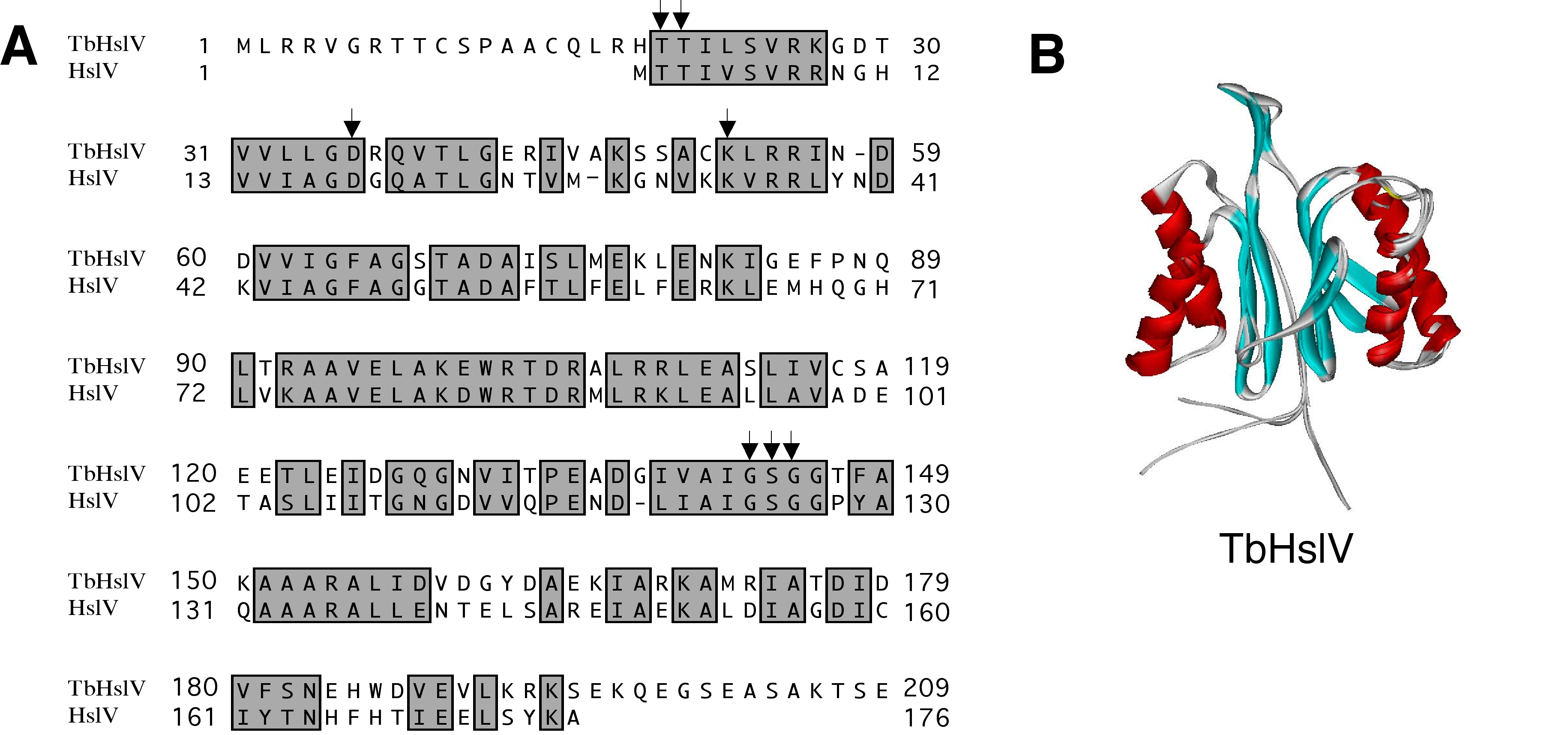

Supplement: Figure S1 — TbHslV resembles E. coli HslV protease. (A). Sequence alignment of TbHslV with HslV. Residues essential for the activity of HslV are indicated by arrows; (B). The homology model of TbHslV. Generation of the three-dimensional models was performed using Swiss-Model (http://swissmodel.expasy.org/) [31] according to the corresponding E. coli templates. The images were then analyzed with Swiss-Pdb-Viewer 3.7 (http://swissmodel.expasy.org/spdbv/). Protein Data Bank codes for the templates of HslV were 1ned [4], le94 [55] and 1hqy [56]. (1.21 MB TIF) [file ppat.1000048.s001.tif]

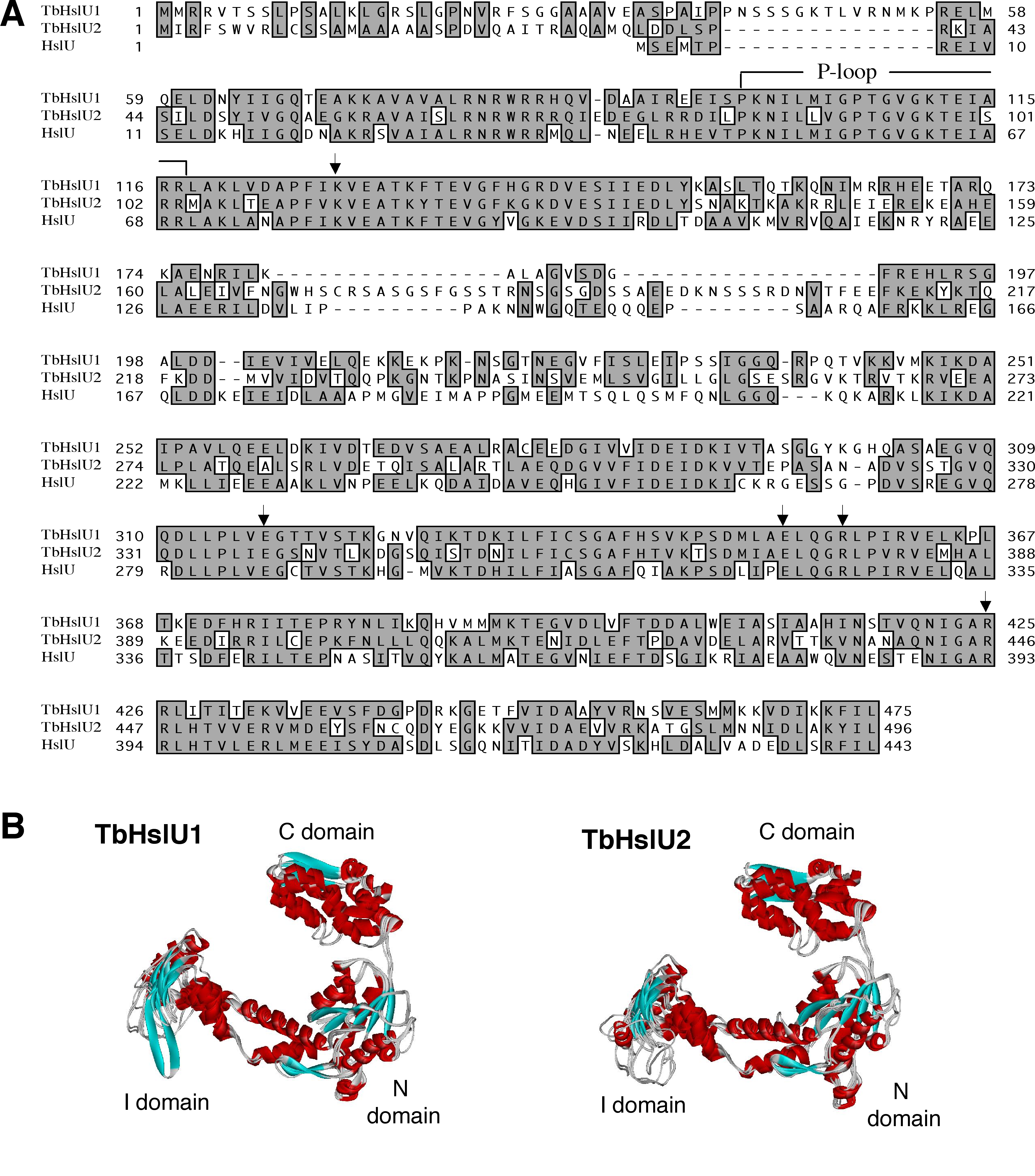

Supplement: Figure S2 — Both TbHslU1 and TbHslU2 resemble E. coli HslU. (A). Sequence alignment of TbHslU1, TbHslU2 with HslU. The NTP-binding domain (P-loop) is outlined and the residues important for HslU function are indicated by arrows; (B) The homology models of TbHslU1 and TbHslU2. Each structure was modeled on an E. coli HslU template. The three domains identified in HslU are also present in the two T. brucei homologs. The protein bank code for the template of HslU is 1do0 [4]. (3.28 MB TIF) [file ppat.1000048.s002.tif]

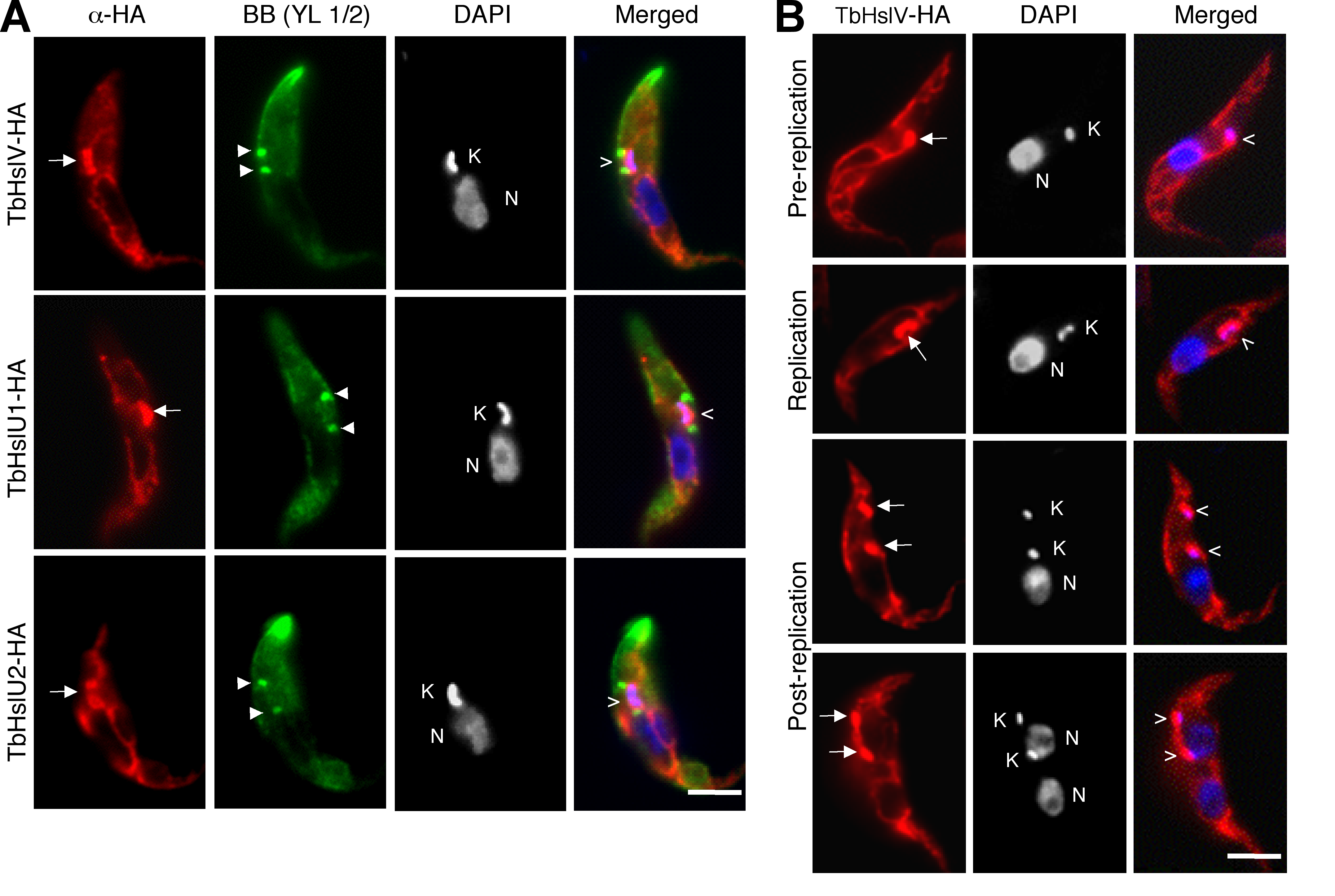

Supplement: Figure S3 — (A). Co-localization of TbHslVU proteins with the kinetoplast. Cells were labeled with anti-HA antibody for TbHslVU-HA (red), YL1/2 antibody for basal body (BB, green), and DAPI for nuclear (N) and kinetoplast (K) DNA. Arrows point to the bright spots of HA labeling, arrowheads point to the basal bodies, and open arrowheads indicate the co-localization of TbHslVU-HA protein with kinetoplasts. Bar: 2 µm. (B). Subcellular localization of TbHslV during different stages of kinetoplast cycle. Cells were labeled with anti-HA antibody for TbHslV-HA, and DAPI for nuclear (N) and kinetoplast (K) DNA. Arrows point to the bright spots of HA labeling, and open arrowheads indicate co-localization of TbHslV-HA with kinetoplasts. Bar: 2 µm. (4.63 MB TIF) [file ppat.1000048.s003.tif]

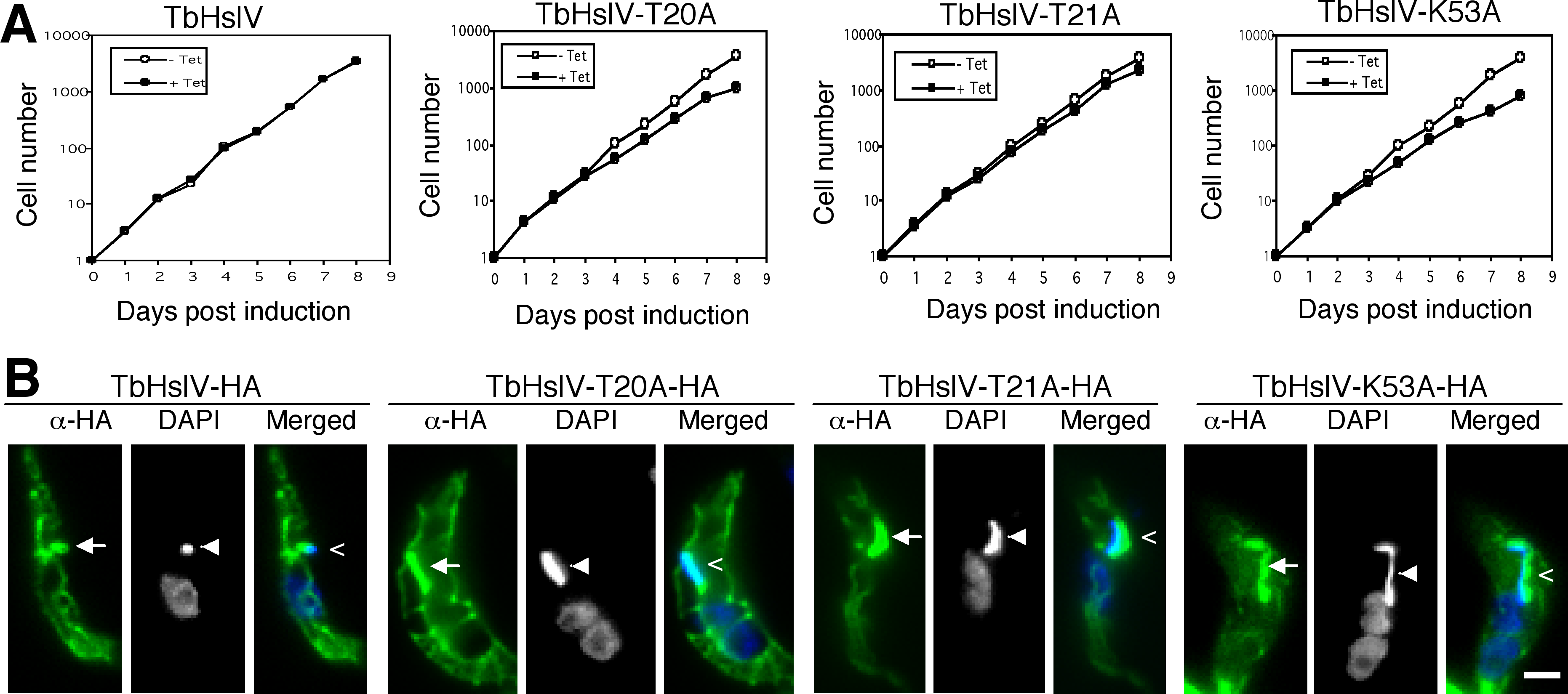

Supplement: Figure S4 — Effects of expressing HA-tagged wild type and mutant TbHslV on cell growth (A) and kinetoplast segregation (B). Cells were labeled with anti-HA antibody (green) and counterstained with DAPI for the nucleus and kinetoplast. The arrows point to the bright spots of HA staining, the solid arrowheads point to the kinetoplasts and the open arrowheads indicate the HA spots superimposed with kinetoplasts. Bar: 2 µm. (0.93 MB TIF) [file ppat.1000048.s004.tif]
